# SUV variability in EARL-accredited conventional and digital PET

**DOI:** 10.1186/s13550-019-0569-7

**Published:** 2019-12-10

**Authors:** Daniëlle Koopman, Pieter L. Jager, Cornelis H. Slump, Siert Knollema, Jorn A. van Dalen

**Affiliations:** 1 Department of Nuclear Medicine, Isala, Dokter van Heesweg 2, 8025 AB Zwolle, the Netherlands; 2 Technical Medicine Center, University of Twente, Enschede, the Netherlands; 3 Department of Medical Physics, Isala, Zwolle, the Netherlands

**Keywords:** *FDG-PET*, *EARL-accreditation*, *Conventional PET*, *Digital PET*, *Cancer*

## Abstract

**Background:**

A high SUV-reproducibility is crucial when different PET scanners are in use. We evaluated the SUV variability in whole-body FDG-PET scans of patients with suspected or proven cancer using an EARL-accredited conventional and digital PET scanner.

In a head-to-head comparison we studied images of 50 patients acquired on a conventional scanner (cPET, Ingenuity TF PET/CT, Philips) and compared them with images acquired on a digital scanner (dPET, Vereos PET/CT, Philips). The PET scanning order was randomised and EARL-compatible reconstructions were applied.

We measured SUV_mean_, SUV_peak_, SUV_max_ and lesion diameter in up to 5 FDG-positive lesions per patient. The relative difference ΔSUV between cPET and dPET was calculated for each SUV-parameter. Furthermore, we calculated repeatability coefficients, reflecting the 95% confidence interval of ΔSUV.

**Results:**

We included 128 lesions with an average size of 19 ± 14 mm. Average ΔSUVs were 6-8% with dPET values being higher for all three SUV-parameters (*p* < 0.001). ΔSUV_max_ was significantly higher than ΔSUV_mean_ (8% vs. 6%, *p* = 0.002) and than ΔSUV_peak_ (8% vs. 7%, *p* = 0.03). Repeatability coefficients across individual lesions were 27% (ΔSUV_mean_ and ΔSUV_peak_) and 33% (ΔSUV_max_) (*p* < 0.001).

**Conclusions:**

With EARL-accredited conventional and digital PET, we found a limited SUV variability with average differences up to 8%. Furthermore, only a limited number of lesions showed a SUV difference of more than 30%. These findings indicate that EARL standardisation works.

**Trial registration:**

This prospective study was registered on the 31th of October 2017 at ClinicalTrials.cov. URL: https://clinicaltrials.gov/ct2/show/NCT03457506?id=03457506&rank=1.

## Background

Positron emission tomography/computed tomography (PET/CT) using fluor-18 fluorodeoxyglucose (FDG) is widely used for tumour imaging in patients with cancer. There are ongoing efforts towards standardisation of FDG-PET imaging to allow a quantitative comparison between patients, scanners and medical centres. In 2009 and 2015 the European Association of Nuclear Medicine (EANM) published procedure guidelines on FDG-PET/CT tumour imaging [1, 2]. Furthermore, the EANM launched the EANM Research Ltd. (EARL) to promote nuclear medicine research, including multi-centre trials. In 2010, EARL started an accreditation program for FDG-PET/CT tumour imaging. This includes EARL-accreditation requirements based on activity concentration recovery coefficients (CRCs) as measured in PET images of a NEMA NU2-2001 image quality phantom. A recent evaluation among the first 200 accredited systems from 150 sites worldwide showed that setting up a harmonising accreditation program is feasible and achievable, and that the FDG-PET/CT program has reduced the variability in semi-quantitative PET performance [3].

Recently, time-of-flight (TOF) PET systems with silicon photomultipliers (SiPM) with digital readout were introduced in clinical practice [4–6]. Although these systems potentially improve image quality compared with PET systems using conventional photomultiplier technology, they can also fulfil EARL accreditation specifications for tumour imaging with FDG-PET/CT when appropriate reconstruction settings are used [6, 7]. Hence, independent of detector technology, PET systems should provide comparable semi-quantitative results once they fulfil EARL specifications. To our knowledge, this has not yet been explored in clinical practice in a substantial group of patients. Therefore, our aim was to investigate the variability in standardised uptake values (SUVs) on whole-body FDG-PET scans from patients with cancer, using both a conventional and digital EARL-accredited PET scanner.

## Materials and methods

### Inclusion

We performed a prospective single-centre side-by-side comparison study in 50 patients with suspected or proven cancer who were referred for whole-body FDG-PET/CT. Written informed consent was obtained from all participants included in this study. The Medical Ethical Committee of our institution (METC Isala, Zwolle, Netherlands) approved the study protocol (NL52329**.**075**.**15).

### PET/CT acquisition

Patients fasted for at least 6 h prior to the PET scan. Blood glucose levels were measured before intravenous injection of FDG, to ensure a value below 10 mmol/L. Patients were administered a FDG-activity based on *A* = 6.2 *w*^2^/*t*, where *A* is the FDG-activity administered in Megabecquerel (MBq), *w* is the patient’s body weight in kilogram (kg) and *t* is the acquisition time per bed position in seconds (s) [8].

For each patient whole-body PET scans from head to groin were acquired in supine position using a state-of-the-art TOF PET/CT scanner with conventional photomultiplier technology (cPET, Ingenuity TF, Philips Healthcare) and a TOF PET/CT scanner with digital SIPMs and digital readout (dPET, Vereos, Philips Healthcare). Both systems were EARL-accredited. For both PET scanners the error in cross-calibration with the associated dose calibrator was less than 5%. The PET scanning order was randomised per patient. We included 25 patients who were first scanned on dPET and afterwards on cPET (*dPET-first group*), and we included 25 patients who were first scanned on cPET and afterwards on dPET (*dPET-second group*). Per patient and per scan we collected ΔT which was defined as the time between FDG-administration and the start of the PET scan.

PET acquisition times of the first scan were 72 s and 144 s per bed position for patients with body weight ≤ 80 kg and > 80 kg, respectively. For the second scan the scan time per bed position was equal to the scan time of the first scan plus a compensation for the radioactive decay of fluor-18. The resulting average scan time of the second PET scan was 85 s (range 72–91 s) for patients ≤ 80 kg and 180 s (range 147–205 s) for patients > 80 kg.

Prior to each PET scan a CT scan was acquired for attenuation correction. The CT scan parameters were 120 kV, 64 mAs (range 39–136 mAs), 64 × 0.625 mm slice collimation, a pitch of 0.83 and a rotation time of 0.5 s.

### PET/CT reconstruction

For both systems we used EARL-compatible reconstructions. For cPET an ordered subset expectation maximisation (OSEM) TOF PET reconstruction was applied with 4 × 4 × 4 mm^3^ voxels and a relaxation parameter of 1.0, without point spread function (PSF) modelling, as previously described [9]. For dPET we performed an OSEM TOF PET reconstruction with 4 × 4 × 4 mm^3^ voxels and a 3-mm Gaussian post-smoothing filter, without PSF modelling, as previously described [7]. For both cPET and dPET attenuation correction was applied using iteratively reconstructed CT data with iDose level 4 and a slice thickness of 3 mm.

### Semi-quantitative evaluation

Semi-quantitative analyses were performed using the quAntitative onCology moleCUlar Analysis suiTE (ACCURATE) tool [10]. For each patient we included a maximum of 5 FDG-positive lesions, to prevent a possible bias from patients with many lesions. In case a patient had more than 5 eligible lesions, we selected the 5 lesions with the shortest diameter on the CT scan and which were measurable on both PET scans using the ACCURATE tool. We chose this selection approach because smaller lesions can be more sensitive to recon differences.

For each lesion we measured the mean, peak and maximum standardised uptake value (SUV_mean_, SUV_peak_ and SUV_max_) on cPET and dPET images. SUV_mean_ was based on the 3D isocontour derived at 50% of the maximum pixel value. SUV_peak_ was defined as the average SUV of a spherical 1 cm^3^ volume-of-interest in the tumour-region with the highest uptake [11]. Furthermore, we measured the short-axis diameter on the axial slice of the CT scan.

Following the paper by Lodge [12] we calculated the relative difference ∆SUV per lesion between cPET and dPET for SUV_mean_, SUV_peak_ and SUV_max_ using Eq. 1.

1 $$ \Delta \mathrm{SUV}=\frac{{\mathrm{SUV}}_{\mathrm{dPET}}-{\mathrm{SUV}}_{\mathrm{cPET}}}{\left({\mathrm{SUV}}_{\mathrm{dPET}}+{\mathrm{SUV}}_{\mathrm{cPET}}\right)\times 0.5} $$

In addition, we derived the standard deviation (SD) of ∆SUV and we calculated the repeatability coefficient (RC) using Eq. 2.

2 $$ \mathrm{RC}=1.96\times \mathrm{SD}\left(\Delta \mathrm{SUV}\right) $$

The RC reflects the 95% confidence interval of ΔSUV. Moreover, we counted the number of lesions with an absolute ΔSUV ≥ 30% for all three SUV-parameters as this cut-off value is considered by PERCIST to indicate a switch from “stable” disease to either “progression” or “response” [13].

### Statistical analysis

The statistical analysis was performed using SPSS Version 24. Quantitative results were presented as mean ± SD. Data distribution normality was evaluated using the Shapiro-Wilk test. For data that were not normally distributed the median was included as well. We performed an independent-sample *t* test to compare patient and scan characteristics (age, body weight, administered FDG-activity and ΔT) between patients in both scanning groups. Furthermore, we performed the Mann-Whitney *U*-test to compare lesion diameters between lesions in both scanning groups. Differences in average SUV_mean_, SUV_peak_ and SUV_max_ between cPET and dPET were evaluated with the Wilcoxon signed-rank test. To test whether average ΔSUV differences between the two PET systems were similar for the three SUV-parameters, we pairwise compared ΔSUV_mean_, ΔSUV_peak_ and ΔSUV_max_ using a paired 2-sample *t* test. Furthermore, we performed the Pitman-Morgan test (using R studio, package PairedData) to pairwise compare the RCs of the three SUV-parameters. Moreover, we performed a linear regression analysis (Pearson’s correlation coefficient and *F*-test) to determine correlations between ΔSUV and the time between FDG-administration and the start of the dPET scan (ΔT_dPET_), and between ΔSUV and lesion diameter. A *p* value less than 0.05 was considered to indicate statistical significance.

## Results

### Patient characteristics

We included 50 patients (27 males, 23 females) with suspected or proven lung cancer (*n* = 35), breast cancer (*n* = 8), lymphoma (*n* = 3), oesophageal cancer (*n* = 3) or gastric cancer (*n* = 1). Patient and scan characteristics per scanning group are presented in Table 1. The characteristics of both groups were comparable (*p* ≥ 0.16). In total we evaluated 128 FDG-positive lesions, among which 66 lesions were part of the dPET-first group and 62 lesions of the dPET-second group. The average lesion diameter was 19 ± 14 mm (median 15 mm, range 4–90 mm) with comparable sizes across both scanning groups (*p* = 0.36). The number of included lesions per patient was 1 in 17 patients, 2 in 11 patients, 3 in 7 patients, 4 in 7 patients and 5 in 8 patients.

**Table 1:** Patient (*n* = 50) and scan characteristics

	dPET-first group (*n* = 25)	dPET-second group (*n* = 25)	*p* value
Age (in years)^a^	64 ± 10	67 ± 12	*0.23*
Body weight (in kg)^a^	83 ± 19	76 ± 16	*0.16*
Glucose level (in mmol/L)^a^	5.7 ± 0.8	6.0 ± 1.0	*0.25*
Administered FDG-activity (in MBq)^a^	413 ± 105	397 ± 97	*0.60*
ΔT from FDG administration			
Until first PET scan (in min)^a^	64 ± 10	66 ± 10	*0.51*
Until second PET scan (in min)^a^	96 ± 11	97 ± 13	*0.79*

^a^Continuous variables are described as mean ± SD

### Semi-quantitative evaluation

**Fig. 1:**
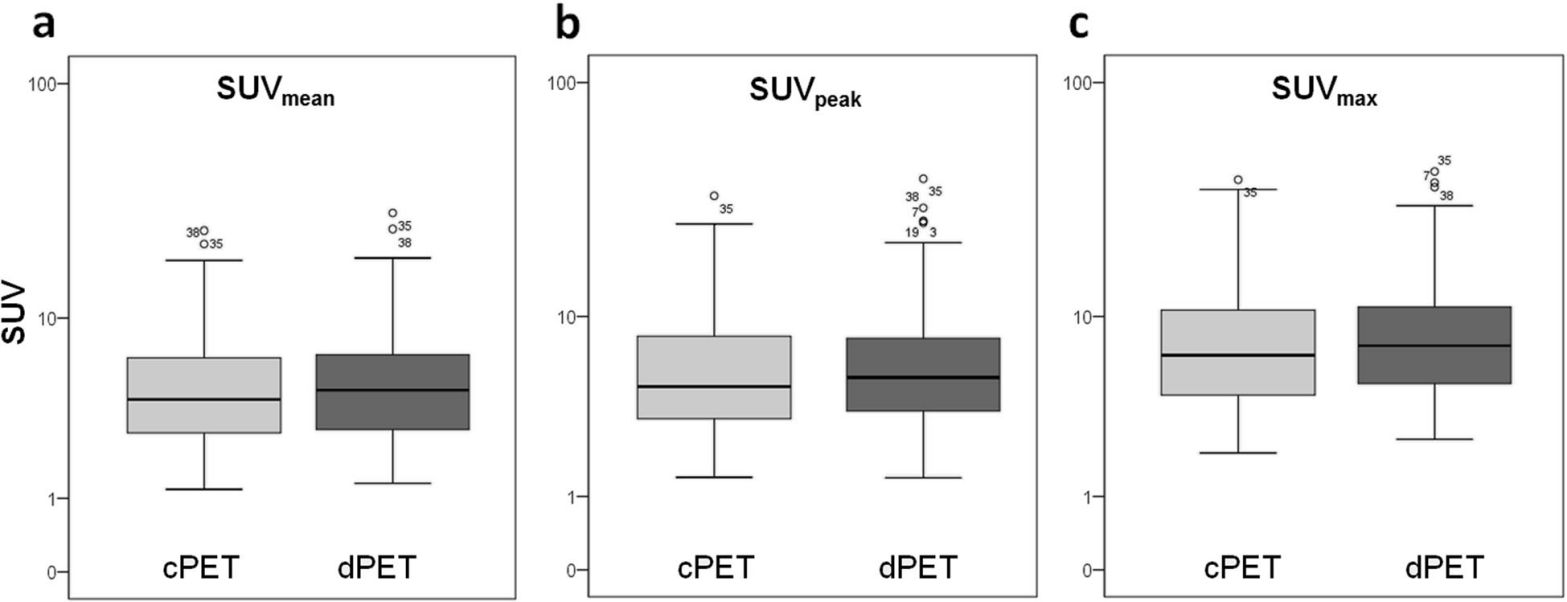
SUV_mean_ (**a**), SUV_peak_ (**b**) and SUV_max_ (**c**) as measured on cPET and dPET across all lesions (*n* = 128). The *y*-axis is shown on a log scale. Average dPET values were significantly higher than cPET values for all three parameters (*p* < 0.001). This boxplot shows the median, interquartile range and outliers (o): values that are between 1.5 and 3.0 box length from the percentile borders

SUV_mean_, SUV_peak_ and SUV_max_ over all 128 lesions are shown in Table 2 and Fig. 1 for cPET and dPET separately. Average dPET values were higher than cPET values for all three SUV-parameters (*p* < 0.001).

**Table 2:** Average SUV_mean_, SUV_peak_ and SUV_max_ across all lesions (*n* = 128), the relative difference ΔSUV between both systems and the RC per SUV-parameter. dPET SUVs were higher than cPETSUVs (*p* < 0.001) with average ΔSUVs of 6–8%

	cPET^a^	dPET^a^	ΔSUV (%)^a^	RC	*p* value
SUV_mean_	5.3 ± 3.8 (4.1)	5.6 ± 4.3 (4.6)	6% ± 14%	*27%*	*< 0.001*
SUV_peak_	6.4 ± 5.2 (4.7)	6.8 ± 5.9 (5.2)	7% ± 14%	*27%*	*< 0.001*
SUV_max_	8.4 ± 6.3 (6.6)	9.1 ± 7.0 (7.3)	8% ± 17%	*33%*	*< 0.001*

^a^Continuous variables are described as mean ± SD (and median if not normally distributed)

**Fig. 2:**
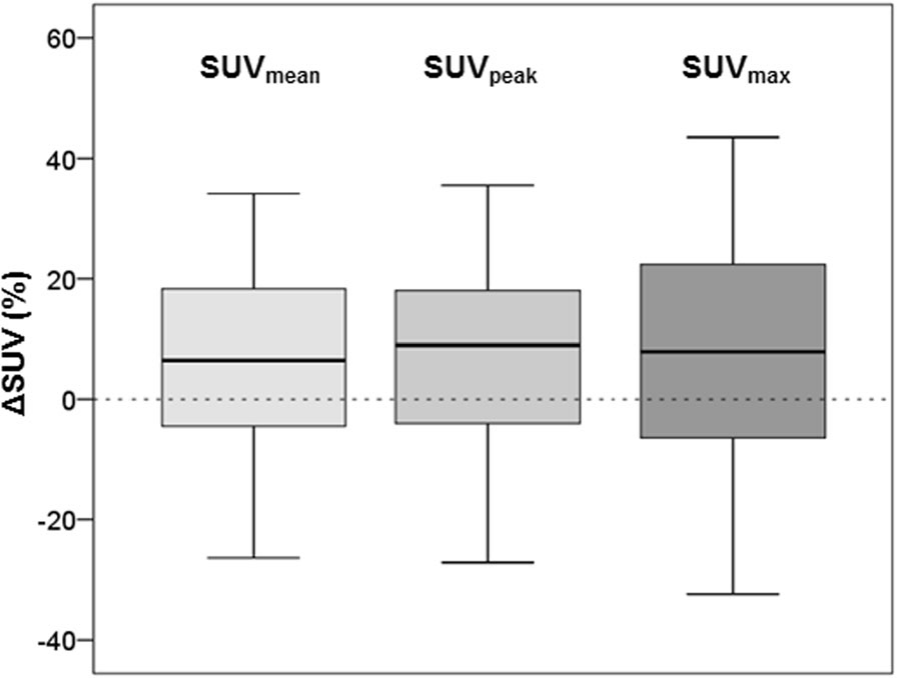
ΔSUV variability for SUV_mean_, SUV_peak_ and SUV_max_ between cPET and dPET across all lesions (*n* = 128). The average variability in ΔSUV_max_ was larger than the variability in ΔSUV_mean_ (*p* = 0.002) and ΔSUV_peak_ (*p* = 0.03). Furthermore, ΔSUV_max_ had a higher variance as compared with ΔSUV_mean_ and ΔSUV_peak_ (*p* < 0.001). This boxplot shows the median and the interquartile range

Furthermore, relative SUV differences (ΔSUV) between cPET and dPET are shown in Table 2 and Fig. 2. The average variability in SUV_max_ was significantly higher than in SUV_mean_ (8% vs. 6%, *p* = 0.002) and in SUV_peak_ (8% vs. 7%, *p* = 0.03), while ΔSUV_mean_ and ΔSUV_peak_ were similar across all lesions (6% vs. 7%, *p* = 0.08). Furthermore, corresponding RCs were 27% (ΔSUV_mean_ and ΔSUV_peak_) and 33% (ΔSUV_max_), with the RC of SUV_max_ being higher than the RCs of SUV_mean_ and SUV_peak_ (*p* < 0.001). SUV_mean_ and SUV_peak_ RCs were similar (*p* = 0.35).

The number of lesions with an absolute ΔSUV ≥ 30% was 3 (2%) for SUV_mean_, 4 (3%) for SUV_peak_ and 15 (12%) for SUV_max_. All lesions but one with a ΔSUV variability of ≥ 30% were part of the dPET-second group.

**Fig. 3:**
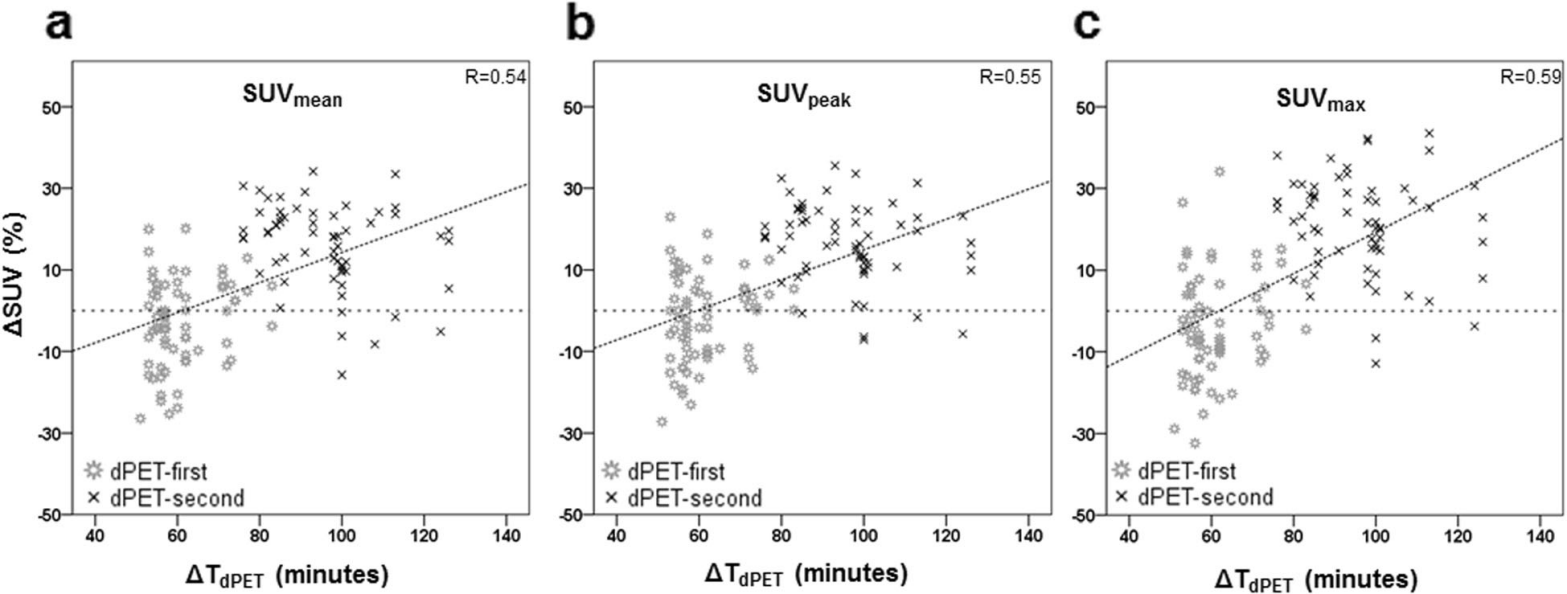
Scatterplot comparing the relative change in SUV_mean_ (**a**), SUV_peak_ (**b**) and SUV_max_ (**c**) with ΔTdPET, defined as the time between FDG-administration and start of the dPET scan. ΔSUV_mean_, ΔSUV_peak_ and ΔSUV_max_ increased with prolonged ΔT_dPET_ (*p* < 0.001)

**Fig. 4:**
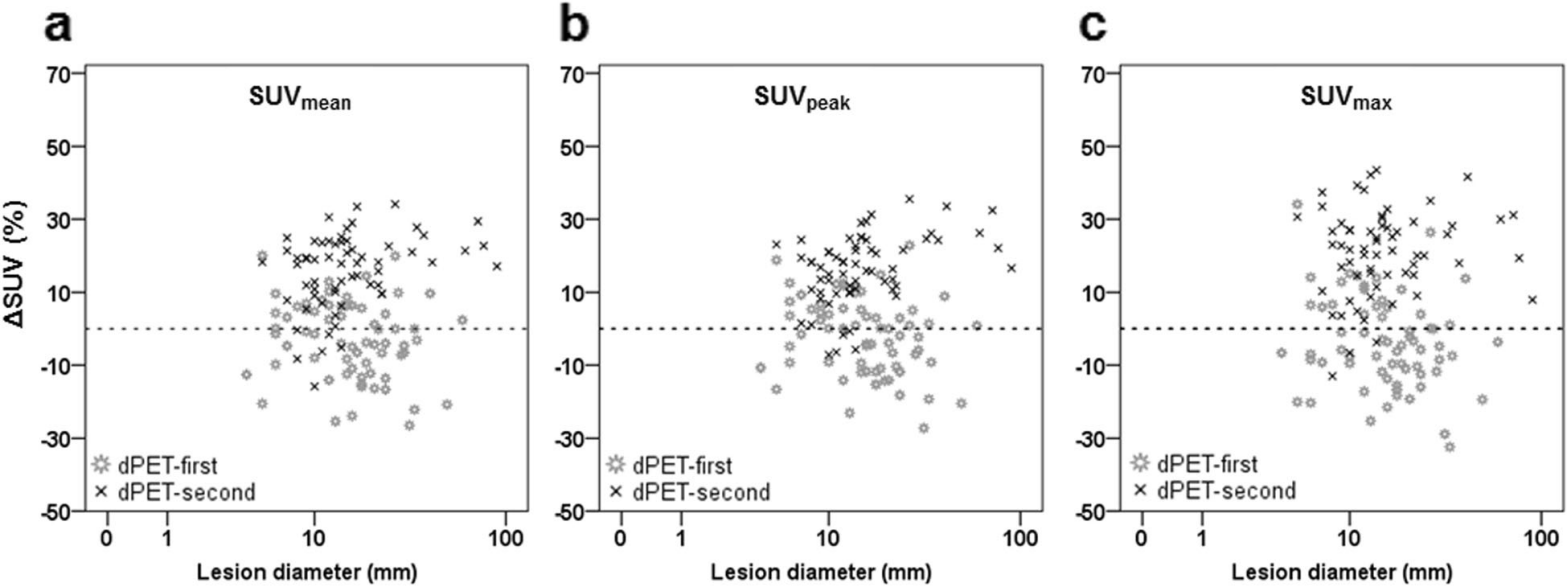
Scatterplot comparing the relative change in SUV_mean_ (**a**), SUV_peak_ (**b**) and SUV_max_ (**c**) with lesion diameter. The *x*-axis is shown on a log scale. There were no significant correlations between ΔSUV and lesion diameter with *R* = 0.09 for ΔSUV_mean_ and ΔSUV_peak_ (*p* = 0.32), and *R* = 0.01 for ΔSUV_max_ (*p* = 0.96)

Correlations between ΔSUV and ΔT_dPET_ are presented in Fig. 3 for all three SUV-parameters. It shows that ΔSUV_mean_, ΔSUV_peak_ and ΔSUV_max_ increased at prolonged ΔT_dPET_ (*p* < 0.001) with correlation coefficients of 0.54, 0.55 and 0.59, respectively. Furthermore, the average ΔSUV of lesions in the dPET-second group was significantly higher as compared with lesions in the dPET-first group, with ΔSUV_mean_ of 16% and − 3%, respectively (*p* < 0.001), ΔSUV_peak_ of 16% and − 2%, respectively (*p* < 0.001), and ΔSUV_max_ of 21% and − 4%, respectively (*p* < 0.001). In Fig. 4 we compared ΔSUV for each lesion with its diameter. We found no correlation between these two parameters (*R* < 0.09, *p* > 0.33).

### Clinical example

**Fig. 5:**
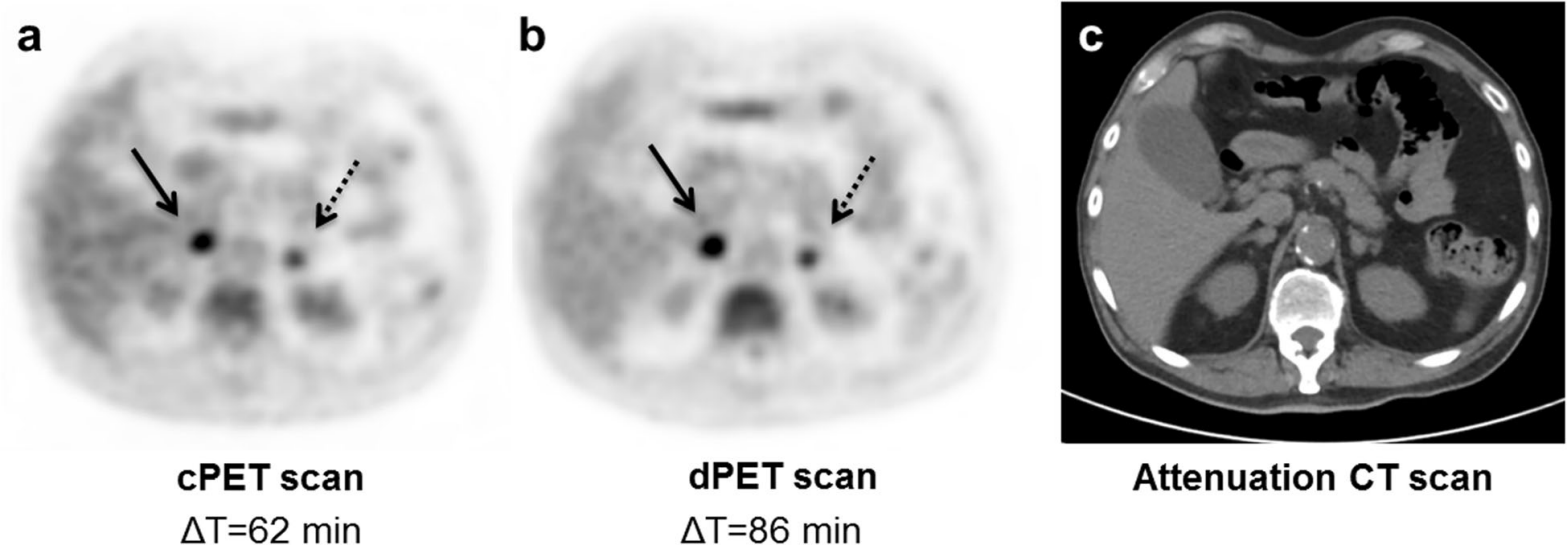
Axial FDG-PET/CT images (**a**, **b** and **c**) from a lung cancer patient with bilateral adrenal gland metastases showing higher SUVs on the dPET scan (**b**) that was acquired 24 min after the cPET scan (**a**). The left-gland metastasis (diameter 11 mm) showed ΔSUVs of 7% (SUV_mean_), 10% (SUV_peak_) and 15% (SUV_max_). ΔSUVs of the right-gland metastasis (diameter 14 mm) were 13% (SUV_mean_) and 11% (SUV_peak_ and SUV_max_). In this case the impact of the digital scanner cannot be separated from the SUV rise caused by the prolonged FDG-uptake. Meanwhile, the visual image quality of both PET scans appeared comparable in terms of image noise, texture and FDG-uptake as intended with an EARL-compatible protocol

In Fig. 5, FDG-PET/CT images are shown from a patient with suspected lung cancer in the dPET-second group. Both PET scans showed bilateral adrenal gland metastases with higher SUVs (ΔSUV 7–15%) on the second dPET scan that was acquired 24 min after the cPET scan.

## Discussion

We evaluated the SUV variability in whole-body FDG-PET scans from 50 patients with cancer by comparing conventional and digital EARL-accredited PET. The average SUV variability across 128 FDG-positive lesions was limited with ΔSUVs of 6–8%. Furthermore, only a limited number of lesions showed a SUV difference of more than 30%. These findings indicate that EARL standardisation works.

We compared the variability of three SUV-parameters in a pairwise fashion, and as expected we found the variability in SUV_max_ to be higher than in SUV_mean_ and SUV_peak_ (*p* ≤ 0.03), although the average differences were relatively small (8% vs. 6–7%)_._ We used automated software to identify the tumour region with the highest uptake within the lesion, and it has been suggested that this method provides a lower variability for SUV_peak_ as compared with SUV_max_ [12]. Recently, EARL adopted SUV_peak_ as an additional metric in the updated EARL accreditation standards [14], as it appeared to be less sensitive to changes in reconstruction parameters and acquisition durations than SUV_mean_ or SUV_max_ [15]. However, a drawback of common SUV_peak_ definitions is that its volume of 1 cm^3^ is not suitable for (sub)centimeter lesions [15].

We found repeatability coefficients of 27% (SUV_mean_ and SUV_peak_) and 33% (SUV_max_). This variability is likely caused by a combination of three factors: a difference in EARL CRCs between our cPET and dPET system, the impact of prolonged FDG-uptake and the SUV test-retest variability. These 3 factors are discussed in the next 3 paragraphs.

Concerning CRC differences, the EARL protocol for our dPET system was based on relatively high CRCs for sub-15 mm small spheres [7] as compared with the CRCs of our cPET EARL protocol [9], with 10–20% higher CRCs on dPET. This explains why we found average ΔSUVs of 6–8% with dPET SUVs being higher than cPET values (*p* < 0.001) in most cases. Larger variations can be expected at other PET sites or in clinical trials that use multiple EARL-accredited PET systems with divergent CRCs. This is possible because current EARL accreditation specifications [16] accept relatively large differences in CRCs, especially for small spheres (Table 3). To further harmonise the semi-quantitative results of EARL-accredited PET scanners, PET reconstruction settings could be further aligned to provide more similar CRCs. Naturally, SUV variability could also be reduced by using the same system and therefore this should be applied in longitudinal PET comparisons of the same patient [17].

**Table 3:** CRC_mean_ and CRC_max_ limits for the six phantom spheres as defined by EARL [17]. For each sphere, relative differences between the upper and lower CRC limits were calculated using ${\mathrm{CRC}}_{\mathrm{dif}}\left(\%\right)=\frac{\mathrm{maximumCRC}-\mathrm{minimumCRC}}{\left(\mathrm{maximumCRC}+\mathrm{minimumCRC}\right)\times 0.5}\times 100$

Sphere diameter	Limits			
	CRC_mean_		CRC_max_	
	min–max limits	CRC_dif_ (%)	min–max limits	CRC_dif_ (%)
10 mm	0.27–0.43	46%	0.34–0.57	51%
13 mm	0.44–0.60	31%	0.59–0.85	36%
17 mm	0.57–0.73	25%	0.73–1.01	32%
22 mm	0.63–0.78	21%	0.83–1.09	27%
28 mm	0.72–0.85	17%	0.91–1.13	22%
37 mm	0.76–0.89	16%	0.95–1.16	20%

Concerning the time-interval between the first and the second scan, it is known that SUVs generally increase with prolonged FDG-uptake [18, 19]. We corrected for this effect by randomising the PET scanning order. Consequently, the average ΔSUV across all lesions is likely not influenced by this effect. However, ΔSUVs of individual lesions were higher after the longer interval as shown in Fig. 3. It is likely that the higher average ΔSUV for lesions in the *dPET-second group* is both caused by an increase in SUV due to prolonged FDG-uptake as well as the effect of the digital scanner with its higher CRCs. Conversely, in the *dPET-first group* an increase in SUV on the second scan caused by prolonged FDG-uptake is partly being compensated as cPET images were based on a reconstruction with lower CRCs as compared with dPET. For example, the average ΔSUV_max_ in the dPET-first group was − 4% whereas the average ΔSUV_max_ in dPET-second group was 21%. Based on these averages, we expect that about (21% + 4%)/2 = 13% of the higher SUV_max_ on the second scan can be attributed to the prolonged FDG-uptake time. If this theoretical correction of 13% is applied to all individual ΔSUVs, only 1 lesion (1%) remains with a ΔSUV_max_ ≥ 30%.

Concerning the SUV test-retest variability, it is known that biological, technical and methodological factors [12, 19] play a role. Several studies have evaluated this in different types of cancer [12, 20–22]. In a recent review, Lodge [12] stated that with a strict protocol, lesion-SUV has a within-subject coefficient of variation (wCV) of 10% (SUV_mean_ and SUV_peak_) and 11% (SUV_max_). In our study, we found RCs of 27–33%, representing wCVs of 10% (SUV_mean_ and SUV_peak_) and 12% (SUV_max_) when using $\mathrm{wCV}=\mathrm{RC}/\left(\sqrt{2}\times 1.96\right)$. This indicates that the average ΔSUV in our study is comparable with values reported by Lodge [12]. However, our study includes two aspects that make it difficult to compare our wCVs with the data reported by Lodge. First, we performed both PET scans on the same day after a single FDG-administration while Lodge [12] only included results based on two separate FDG-administrations. Second, the lesions that we included were relatively small (median size 15 mm) while Lodge [12] described that most repeatability studies included lesions with a minimum diameter of 20 to 30 mm.

Our conclusion that EARL standardisation works is in agreement with findings from a recently published paper by van Sluis et al. [23]. They performed a cPET versus dPET comparison study, using scanners from another vendor, in a small group of patients with cancer (*n* = 20). Although they did not calculate relative differences or repeatability coefficients, they observed a good agreement in SUV measurements between both PET/CT systems, in particular when using EARL-compliant reconstructions on both systems [23].

The present study has some limitations. We included 128 lesions across 50 patients where the included number of lesions varied between 1 and 5 lesions per patient, but we did not take a possible intra-patient correlation between lesions into account in the statistical analysis. Yet, the number of lesions in both scanning groups was almost similar (66 vs. 62 lesions). Furthermore, our study was not a full test-retest study since for each patient both PET scans were acquired on the same day and with just a single FDG-injection. Therefore, variability associated with patient preparation, biological factors and FDG-administration was not fully taken into account in our study. However, other factors such as patient motion, breathing and potential CT-PET mismatches could still have influenced the ΔSUV in this intra-individual comparison of EARL-accredited cPET and dPET scans. Still, given that the impacts of the PET systems, biological effect and test-retest are intricate and that biological effects are not negligible, it would be useful to repeat this semi-quantitative comparison of EARL-accredited PET scans in a full test-retest setting to confirm our results. Another limitation is the wide range in ΔT for the second scan as shown in Fig. 3, which influences individual ΔSUVs. Fortunately, the average FDG-uptake time per scan between both scanning groups was similar.

While the present study is based on current EARL accreditation specifications [16], an update of those specifications has been proposed because in recent years different vendors launched new PET/CT systems equipped with novel techniques such as TOF, resolution modelling/PSF technologies and digital detectors. These modern systems can deliver PET images with higher CRCs, especially for small spheres, and therefore, an update of the EARL accreditation specifications is desirable. Kaalep et al. [15] evaluated the feasibility of harmonising performance for novel PET/CT systems, and they also proposed new EARL criteria. In these newly proposed CRCs the relative difference (%) between upper and lower limits is similar to current EARL specifications [16]. Therefore, it is expected that the potential variability in semi-quantitative FDG-PET with such updated EARL-compatible protocols will remain similar.

## Conclusion

With EARL-accredited conventional and digital PET, we found a limited SUV variability with average differences up to 8%. Furthermore, only a limited number of lesions showed a SUV difference of more than 30%. These findings indicate that EARL standardisation works. When EARL-accredited systems with divergent CRCs are used, larger SUV differences can be expected.

## Data Availability

The datasets used and/or analysed during the current study are available from the corresponding author on reasonable request. Ethics approval and consent to participate

## Abbreviations

ΔT: Time between FDG-administration and start of the PET scan
CRC: Contrast recovery coefficient
CT: Computed tomography
EANM: European Association of Nuclear Medicine
EARL: EANM Research Ltd
FDG: Fluor-18 fluorodeoxyglucose
MIP: Maximum intensity projection
OSEM: Ordered subset expectation maximisation
PET: Positron emission tomography
PSF: Point spread function
RC: Repeatability coefficient
SD: Standard deviation
SUV: Standardised uptake value
SUV_max_: Maximum SUV
SUV_mean_: Average SUV
SUV_peak_: Peak SUV
TOF: Time-of-flight
_W_CV: Within-subject coefficient of variation
